# Head Circumference of Infants Born to Mothers with Different Educational Levels; The Generation R Study

**DOI:** 10.1371/journal.pone.0039798

**Published:** 2012-06-29

**Authors:** Selma H. Bouthoorn, Frank J. van Lenthe, Anita C. S. Hokken-Koelega, Henriëtte A. Moll, Henning Tiemeier, Albert Hofman, Johan P. Mackenbach, Vincent W. V. Jaddoe, Hein Raat

**Affiliations:** 1 The Generation R Study Group, Erasmus Medical Centre, Rotterdam, the Netherlands; 2 Department of Public Health, Erasmus Medical Centre, Rotterdam, the Netherlands; 3 Department of Pediatrics, Erasmus Medical Centre, Rotterdam, the Netherlands; 4 Department of Epidemiology, Erasmus Medical Centre, Rotterdam, the Netherlands; 5 Department of Child and Adolescent Psychiatry, Erasmus Medical Centre, Rotterdam, the Netherlands; Aga Khan University, Pakistan

## Abstract

**Objective:**

Head circumference (HC) reflect growth and development of the brain in early childhood. It is unknown whether socioeconomic differences in HC are present in early childhood. Therefore, we investigated the association between socioeconomic position (SEP) and HC in early childhood, and potential underlying factors.

**Methods:**

The study focused on Dutch children born between April 2002 and January 2006 who participated in The Generation R Study, a population-based prospective cohort study in Rotterdam, the Netherlands. Maternal educational level was used as indicator of SEP. HC measures were concentrated around 1, 3, 6 and 11 months. Associations and explanatory factors were investigated using linear regression analysis, adjusted for potential mediators.

**Results:**

The study included 3383 children. At 1, 3 and 6 months of age, children of mothers with a low education had a smaller HC than those with a high education (difference at 1 month: −0.42 SD; 95% CI: −0.54,−0.30; at 3 months: −0.27 SD; 95% CI −0.40,−0.15; and at 6 months: −0.13 SD; 95% CI −0.24,−0.02). Child’s length and weight could only partially explain the smaller HC at 1 and 3 months of age. At 6 months, birth weight, gestational age and parental height explained the HC differences. At 11 months, no HC differences were found.

**Conclusion:**

Educational inequalities in HC in the first 6 months of life can be mainly explained by pregnancy-related factors, such as birth weight and gestational age. These findings further support public health policies to prevent negative birth outcomes in lower socioeconomic groups.

## Introduction

Growth in childhood is an important indicator of children’s health [1], [2]. Children of parents with low socioeconomic positions (SEP) are found to be smaller, both pre- and postnatal, than children of high SEP parents [2]–[5]. Nutrition, genetic and environmental factors, e.g. maternal smoking, birth weight and maternal height are important mediating factors that may explain these inequalities [4], [6]. There is, however, a need to further improve the understanding of the mechanisms through which SEP affects growth in childhood.

Several studies recognized head circumference (HC) as an important reflection of growth and development of the brain, especially in early childhood [7], [8]. Smaller HC may be associated with a lower intelligence quotient (IQ) and learning problems [8], [9]. This association was even found for HC values immediately under the mean [8]. Lower IQ is related to higher mortality, and SEP has been suggested to be a mediator of this IQ-related mortality [10]. HC is also a sensitive anthropometric indicator of prolonged malnutrition during infancy, so clinicians use HC as a measure of failure to thrive [11], [12].

The effect of SEP on growth of HC has been previously described [4], [13], [14]. A British study found a higher SEP to be associated with greater HC growth in children from 9 months to 9 years [14]. A study from Pakistan also found HC to vary directly with SEP in infancy [13]. Maternal educational level is one of the most frequently used indicators of SEP and has been shown to be a consistent socioeconomic predictor of health [15]–[17]. Furthermore, educational level has been shown to be a good predictor of pregnancy outcomes [18].Therefore, we hypothesized that a low maternal educational level, as indicator of SEP, is associated with smaller HC in childhood. The underlying pathways through which SEP affects HC are not well considered in studies including a broad range of explanatory variables. Thus, the aim of this study was to investigate the association between maternal educational level and HC from birth up to the first year of life. Our second aim was to investigate the possible explanatory mechanisms underlying this association using multivariate regression models.

## Methods

### Study Design and Population

This study was embedded within the Generation R Study, a population-based prospective cohort study from fetal life until young adulthood that has previously been described in detail [19], [20]. Enrollment was aimed in early pregnancy (gestational age <18.0 weeks) at the routine fetal ultrasound examination but was allowed until birth of the child. All children were born between April 2002 and January 2006 and lived in the study area of Rotterdam, The Netherlands (participation rate 61%) [20]. The study was conducted in accordance with the guidelines proposed in the World Medical Association Declaration of Helsinki and has been approved by the Medical Ethical Committee of the Erasmus MC, University Medical Centre Rotterdam. Written consent was obtained from all participating parents.

**Figure 1:**
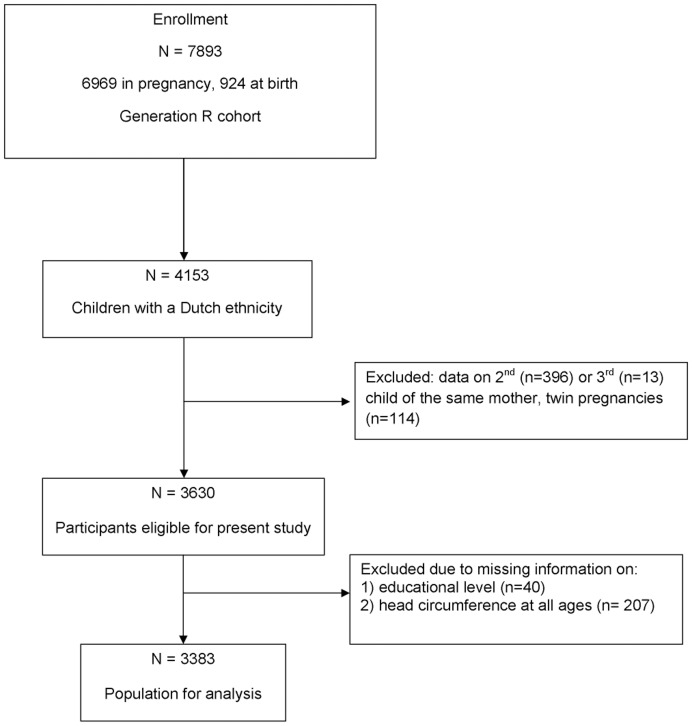
Flowchart of the study population.

Consent of postnatal follow-up was available for 7893 children. We restricted our analyses to the subgroup of children of Dutch ethnicity, because SEP may interact with ethnicity regarding their effects on growth and health [21], [22], [23]. We excluded twins (n = 114), and the second or third child (n = 409) of the same mother, since data were correlated. We also excluded participants without information on maternal educational level (n = 40) and those without HC measurements (n = 207), leaving a study population of 3383 children (Figure 1). Women whose children were excluded from our study were older, more frequently nulliparous at enrollment, less inclined to smoke during pregnancy and had lower psychopathology scores as compared to women whose children were included. The excluded children had a shorter gestational age, were smaller in length, less heavy and had smaller HC’s at 1 and 3 months of age (Table S1).

### Maternal Educational Level

Level of maternal education was established using questionnaires at enrollment. The Dutch Standard Classification of Education was used to categorize 4 subsequent levels of education: 1. high (university degree), 2. mid-high (higher vocational training, Bachelor’s degree), 3. mid-low (>3 years general secondary school, intermediate vocational training) and 4. low (no education, primary school, lower vocational training, intermediate general school, or 3 years or less general secondary school) [20].

### Measurement of Head Circumference

HC measurements were taken during routine screenings at 1, 2, 3, 4, 6 and 11 months by well-trained staff. HC was measured to the nearest 0.1 cm by a tape line. Values were expressed as age- and gender-adjusted standard-deviation scores (SDS) using Dutch reference growth curves [24], [25]. The difference of 1 HC SDS in children under the age of 1 reflects about 1 centimeter [25].

### Potential Mediators

Any effect of maternal educational level on child’s HC is probably an indirect one, acting through more proximal determinants of early growth, so-called mediators [26], [27]. We considered the following factors to be such potential mediators in the pathway between maternal educational level and HC. These were chosen based on previous literature of determinants of child’s HC [5], [18], [28]–[32].

#### Pregnancy and birth characteristics

Birth weight and gestational age at birth were obtained from midwife and hospital registries. We used gestational-age adjusted standard deviation scores for birth weight.

Gestational diabetes was diagnosed according to Dutch midwifery and obstetric guidelines using the following criteria: random glucose level >11.0 mmol/L, fasting glucose >7.0 mmol/l or a fasting glucose between 6.1 and 6.9 mmol/L with a subsequent abnormal glucose tolerance test, in women with no pre-existing diabetes.

Information about smoking and alcohol consumption was assessed in first, second and third trimester. Smoking and alcohol use at enrollment were assessed by a single closed question with three answer options (no, first trimester only, continued in pregnancy). To assess smoking in second and third trimester, mothers were asked whether they smoked in the past 2 months (no, yes) in the second and third questionnaire.

#### Parental anthropometrics

Maternal and paternal heights were measured at our research centers. Pre-pregnancy weight was established at enrollment through questionnaire. On the basis of height and pre-pregnancy weight (weight/height^2^), we calculated pre-pregnancy body mass index (BMI).

#### Psychosocial and material factors

Using questionnaires in early pregnancy, we established whether the pregnancy was planned (yes/no) and the presence of financial difficulties (yes/no).

#### Child characteristics

Because HC, length and weight are related to each other, we evaluated the contribution of the children’s weight and length at time of HC measurement [28]. Standard-deviation scores (SDS) adjusted for age and gender were constructed for all these growth measurements [25]. Information on breastfeeding (yes/no) was derived from questionnaires at the child’s age of 2, 6 and 12 months.

#### Confounding variables

We treated maternal age at enrollment and parity as potential confounders, since they cannot be considered indisputable mediators [26]. Parity was obtained through a questionnaire at enrollment.

### Statistical Analyses

Because head circumference measurements were concentrated around the ages of 1, 3, 6 and 11 months, we assessed the association between mother’s educational level and child’s head circumference at 1 (mid-90% range 0.9–1.4), 3 (mid-90% range 3.0–3.8), 6 (mid-90% range 5.8–6.9) and 11 (mid-90% range 10.4–11.9) months of age using multiple linear regression. Unstandardized regression coefficients, reflecting the difference in HC (in SDS), and 95% confidence intervals (CI) were reported for each educational level compared to the reference category (highest educational level). We started with a model that included the confounders (model 1). Next, this model was additionally adjusted for the potential mediators. For each adjustment, the corresponding percentages of change in HC differences (effect estimates) were calculated by comparing the HC differences of model 1 with the adjusted ones (100 x (B _model 1_– B _model 1 with mediator_)/(B_ model 1_)) (Table S2) [33]. Only those variables that individually produced at least 10% change were added to linear regression models, first separately, then simultaneously (full model). We repeated the analysis including all covariates, and found essentially similar results as compared to the models with only the 10% change covariates included (data not shown). For each covariate, an interaction term with educational level was tested for significance, none of them were significant (data not shown).

Linear mixed models (‘PROC MIXED’ procedure in SAS) were used to assess the association between maternal educational level and longitudinally measured SD scores of HC in the first year of life. In total, we had 16958 measurements of SD scores of HC. The best fitting model structure was: head circumference (in SDS)  =  ß_0_+ ß1 * educational level + ß_2_ * age + ß_3_* educational level * age. In this model the interaction term educational level * age was added with a significance of p<0.001. Age reflects the time of HC measurement.

Percentages of missing values in the covariates ranged from 0% to 36.2% (Table 1). Because the missing values were not completely at random, the multiple imputation procedure in SPSS 17.0 was used [34]. No differences in results were observed between analyses with imputed missing data or complete cases only. Statistical analyses were performed using Statistical Package of Social Science (SPSS) version 17.0 for Windows (SPSS Inc, Chicago, IL, USA) and Statistical Analysis Software (SAS) version 9.2 for Windows (SAS Institute, Cary, NC, USA). A p-value of <0.05 was taken to indicate statistical significance.

**Table 1:** General characteristics of the study population (n = 3383)^a^.

						Maternal educational level							
	Total n = 3383		High n = 1122 (33.2%)		Mid-high n = 871 (25.7%)		Mid-low n = 895 (26.4%)		Low n = 495 (14.6%)		P-value^b^		
**Pregnancy and birth characteristics** c													
Maternal age (years)	31.3 (4.6)		33.1 (3.2)		32.1 (3.9)		30.1 (4.8)		28.0 (5.7)		<0.001		
Parity (% nullipara)	66.1		63.8		68.3		68.7		63.0		0.072		
Infant gender (% girls)	49.8		49.5		50.4		50.6		48.1		0.801		
Gestational age at birth (weeks)	39.9 (1.7)		40.1 (1.6)		40.0 (1.6)		39.8 (2.0)		39.7 (1.6)		<0.001		
Birth weight (grams)	3482.6 (554.3)		3553.9 (534.5)		3514.9 (537.8)		3433.3 (581.1)		3353.5 (548.0)		<0.001		
Gestational diabetes (% yes)	0.8		0.8		0.6		0.7		1.2		0.607		
Maternal smoking during pregnancy (%)													
None	75.7		85.7		79.0		71.7		54.5		<0.001		
Until confirmed pregnancy	10.0		7.9		11.1		10.8		10.9				
Continued during pregnancy	14.3		6.3		9.9		17.4		34.5				
Maternal alcohol use during pregnancy (%)													
None	32.3		18.6		28.4		42.0		52.5		<0.001		
Until confirmed pregnancy	16.8		14.3		18.7		18.8		15.6				
Continued during pregnancy	50.9		67.1		52.9		39.2		31.7				
**Parental anthropometrics** c													
Maternal height (cm)	170.4 (6.5)		171.1 (6.1)		171.0 (6.4)		170.0 (6.6)		168.1 (6.8)		<0.001		
Pre-pregnancy BMI mother (kg/m^2^)		23.1 (3.8)		22.7 (3.1)		22.7 (3.4)		23.6 (4.4)		23.9 (4.7)		<0.001	
Paternal height (cm)		183.9 (7.0)		185.0 (6.7)		184.1 (6.9)		183.6 (7.1)		181.8 (7.3)		<0.001	
**Psychosocial and material factors** c													
Financial difficulties (% yes)		14.4		6.1		12.5		18.4		29.3		<0.001	
Pregnancy planned (% no)		19.1		11.4		14.8		23.1		36.8		<0.001	
**Child characteristics** c													
Length SDS at 1 month of age		−0.15 (1.1)		−0.02 (1.0)		−0.07 (1.0)		−0.21 (1.1)		−0.47 (1.2)		<0.001	
Length SDS at 3 months of age		0.05 (1.0)		0.10 (1.0)		0.04 (1.0)		0.06 (1.0)		−0.08 (1.1)		0.058	
Length SDS at 6 months of age		−0.02 (0.9)		0.01 (0.9)		−0.05 (0.9)		−0.02 (1.0)		−0.01 (2.8)		0.618	
Length SDS at 11 months of age		−0.14 (0.9)		−0.15 (0.9)		−0.17 (0.9)		−0.11 (0.9)		−0.08 (0.9)		0.216	
Weight SDS at 1 month of age		0.09 (1.2)		0.21 (1.1)		0.18 (1.3)		0.0 (1.3)		−0.20 (1.2)		<0.001	
Weight SDS at 3 months of age		0.25 (1.0)		0.30 (1.0)		0.27 (1.0)		0.22 (1.1)		0.15 (1.0)		0.033	
Weight SDS at 6 months of age		0.07 (0.9)		0.07 (0.9)		0.04 (0.9)		0.08 (1.0)		0.10 (0.9)		0.605	
Weight SDS at 11 months of age		−0.02 (0.9)		−0.02 (0.8)		−0.04 (0.9)		−0.02 (0.9)		0.0 (1.0)		0.824	
Breastfeeding (yes %)		90		96		93.7		84.8		79.6		<0.001	

BMI = body mass index, SDS = standard deviation scores.

a Values are percentages or means (SD) for the total population and by level of maternal education.

b P-values are calculated with the Chi-square test for categorical variables and ANOVA for continuous variables.

c Data were missing for parity (2.4%), gestational age (0.1%), smoking during pregnancy (14.5%), alcohol use during pregnancy (14.1%), gestational diabetes (3.3%), maternal height (7.9%), pre-pregnancy BMI (20%), paternal height (17.0%), psychopathology (18.2%), financial difficulties (9.9%), pregnancy planned (5.2%),height SDS at 1 (30.9%), 3 (36.2%), 6 (17.0%) and 11(14.4%) months, weight at 1 (18.0%), 3 (25.9%), 6 (6.7%) and 11 (14.2%) months and breastfeeding (5.7%).

## Results

### Study Population Characteristics

Of the 3383 children, 33.2% of their mothers had a high educational level and 14.6% had a low educational level. Compared with mothers with a high education, those with a low education were on average younger, shorter, more likely to smoke and less likely to drink alcohol during pregnancy. Fewer of them started and continued breastfeeding, they had higher psychopathology scores, more of them had financial difficulties, more had unplanned pregnancies (p<0.001) and more of them suffered from preeclampsia (p = 0.030). Their children were on average lighter at birth and had a shorter gestational duration, a shorter height at 1 month and a lower weight at 1 month (p<0.001) and at 3 months (p = 0.033) of age (Table 1).

Significant differences were observed in mean HC SDS per educational level at 1, 3, 6 and 11 months. Post hoc analyses showed that children from mothers with the highest educational level had significantly larger HCs than children from mothers with mid-low and low educational levels at all ages (Table 2).

**Table 2:** Head circumference SDS characteristics in the total study population and by level of maternal education (n = 3383).

				Maternal educational level									
	Total (n = 3383)		High (n = 1122)		Mid-high (n = 871)		Mid-low (n = 895)		Low (n = 495)		P-value^a^		
**Mean head circumference SDS (SD)**													
1 month missings (%)	0.19 (0.95) 20.2		0.32 (0.90) 19.5		0.25 (0.90) 18.3		0.13 (0.95) 23.8		−0.11 (1.07) 18.8		<0.001		
3 months missings (%)		0.04 (0.88) 27.4		0.15 (0.89) 26.0		0.05 (0.80) 25.4		0.01 (0.92) 31.3		−0.15 (0.92) 27.1		<0.001	
6 months missings (%)	−0.07 (0.89) 8.5		0 (0.86) 7.9		−0.06 (0.85) 8.6		−0.13 (0.92) 8.4		−0.17 (0.95) 9.9		0.001		
11 months missings (%)	−0.07 (0.89) 16.3		0 (0.86) 14.5		−0.06 (0.85) 15.8		−0.10 (0.88) 16.5		−0.13 (0.99) 20.6		0.024		

HC  =  head circumference, SDS  =  standard deviation score.

a Values are calculated with ANOVA for continuous variables and reflect means (SD) per educational level.

### Contribution of Potential Mediators

At 1 and 3 months of age, the differences in HC for the low and/or the mid-low education group were attenuated with more than 10% by individual adjustment for maternal and paternal height, pre-pregnancy BMI, birth weight, gestational age, child’s weight and height and breastfeeding (Table S2). Birth weight and gestational age, when added together to model 1, explained about half of the effect of low education. Adjustment for length and weight explained approximately 60% and 30%, respectively. In the full model, children in the lowest educational subgroup still had significantly smaller HCs at these ages (p = 0.003 and p = 0.012). The association between mid-low education and HC disappeared due to mediation of birth weight and gestational age. At 3 months, adjustment for parental and maternal height had the same effect in the mid-low subgroup (Table 3).

**Table 3:** Differences in child’s head circumference at 1, 3, 6 and 11 months of age between maternal educational levels^a^.

	Maternal educational level			
Models	High education	Mid-high education	Mid-low education	Low education
**1 month of age (n = 2699)**				
Model 1^b^	Reference	−0.07 (−0.17,0.02)	−**0.17 (**−**0.27,** −**0.07)** **	−**0.42 (**−**0.54,** −**.30)** **
Model 1+ paternal and maternal height	Reference	−0.06 (−0.15,0.04)	−**0.12 (**−**0.22,** −**0.03)** *	−**0.32 (**−**0.44,** −**0.20)** **
Model 1+ pre-pregnancy BMI	Reference	−0.08 (−0.17,0.02)	−**0.19 (**−**0.29,** −**0.09)** **	−**0.44 (**−**0.57,** −**0.32)** **
Model 1+ birth weight SDS + gestational age	Reference	−0.04 (−0.11,0.04)	−0.05 (−0.12,0.03)	−**0.18 (**−**0.27,** −**0.08)** **
Model 1+ child’s height and weight SDS at 1 month	Reference	−0.06 (−0.14,0.01)	−**0.08 (**−**0.16,** −**0.001)** *	−**0.16 (**−**0.26,** −**0.07)** **
Model 1+ breastfeeding	Reference	−0.07 (−0.16,0.03)	−**0.15 (**−**0.25,** −**0.05)** **	−**0.38 (**−**0.50,** −**0.26)** **
Fully adjusted model^c^	Reference	−0.06 (−0.13,0.01)	−0.06 (−0.13,0.02)	−**0.15 (**−**0.24,** −**0.05)** **
**3 months of age (n = 2455)**				
Model 1^b^	Reference	−**0.10 (**−**0.19,** −**0.01)** *	−**0.11 (**−**0.21,** −**0.02)** *	−**0.27 (**−**0.40,** −**0.15)** **
Model 1+ paternal and maternal height	Reference	−0.08 (−0.17,0.01)	−0.08 (−0.18,0.01)	−**0.20 (**−**0.31,** −**0.08)** **
Model 1+ pre-pregnancy BMI	Reference	−**0.10 (**−**0.19,** −**0.01)** *	−**0.13 (**−**0.22,** −**0.03)** *	−**0.29 (**−**0.41,** −**0.17)** **
Model 1+ birth weight SDS + gestational age	Reference	−**0.08 (**−**0.16,** −**0.003)** *	−0.05 (−0.14,0.03)	−**0.13 (**−**0.23,** −**0.02)** *
Model 1+ child’s height and weight SDS at 3 months	Reference	−0.07 (−0.14,0.003)	−**0.08 (**−**0.16,** −**0.001)** *	−**0.18 (**−**0.28,** −**0.08)** **
Model 1+ breastfeeding	Reference	−**0.09 (**−**0.19,** −**0.003)** *	−**0.10 (**−**0.20,** −**0.003)** *	−**0.25 (**−**0.37,** −**0.13)** **
Fully adjusted model^c^	Reference	−0.07 (−0.14,0.01)	−0.06 (−0.14,0.02)	−**0.13 (**−**0.23,** −**0.03)** *
**6 months of age (n = 3095)**				
Model 1^b^	Reference	−0.06 (−0.15,0.02)	−**0.11 (**−**0.19,** −**0.02)** *	−**0.13 (**−**0.24,** −**0.02)** *
Model 1+ paternal and maternal height	Reference	−0.05 (−0.13,0.03)	−0.07 (−0.16,0.01)	−0.04 (−0.15,0.07)
Model 1+ pre-pregnancy BMI	Reference	−0.07 (−0.15,0.02)	−**0.12 (**−**0.21,** −**0.03)** **	−**0.15 (**−**0.25,** −**0.04)** **
Model 1+ birth weight SDS + gestational age+ smoking in pregnancy	Reference	−0.04 (−0.12,0.03)	−0.04 (−0.12,0.04)	0.01 (−0.09,0.11)
Fully adjusted model^d^	Reference	−0.04 (−0.12,0.04)	−0.04 (−0.12,0.04)	0.03 (−0.08,0.13)
**11 months of age (n = 2832)**				
Model 1^b^	Reference	−0.06 (−0.15,0.03)	−0.08 (−0.17,0.01)	−0.10 (−0.22,0.01)

* p-value <0.05,

** p-value <0.01,

SDS  =  standard-deviation score, BMI  =  body mass index.

a Values are regression coefficients (95% confidence interval) and reflect the differences in head circumference (in standard deviation scores) in offspring of mothers with mid-high, mid-low and low educational level relative to children of women with high educational level. The values are derived from linear regression analyses performed on the data after multiple imputation of the covariates.

b Model 1: adjusted for maternal age and parity.

c Fully adjusted model: adjusted for parity, maternal age, child’s height and weight (in SDS) at measurement of HC, birth weight SDS, gestational age, paternal and maternal height, pre-pregnancy BMI and breastfeeding (yes/no).

d Fully adjusted model: adjusted for parity, maternal age, birth weight SDS, gestational age, smoking during pregnancy, paternal and maternal height and pre-pregnancy BMI.

At 6 months, an attenuation of 10% was observed after individual adjustment for maternal and paternal height, pre-pregnancy BMI, smoking during pregnancy, birth weight and gestational age (Table S2). When added to model 1, complete elimination of the association of low and mid-low education was observed after adjustment for birth weight, gestational age and smoking during pregnancy and after adjusting for paternal and maternal height. High pre-pregnancy BMI was positively associated with HC SDS at all ages (Table 3 and Table S2). At 11 months of age, there were no differences in HC between the educational subgroups after adjusting for confounders (p>0.05) (Table 3).

**Figure 2:**
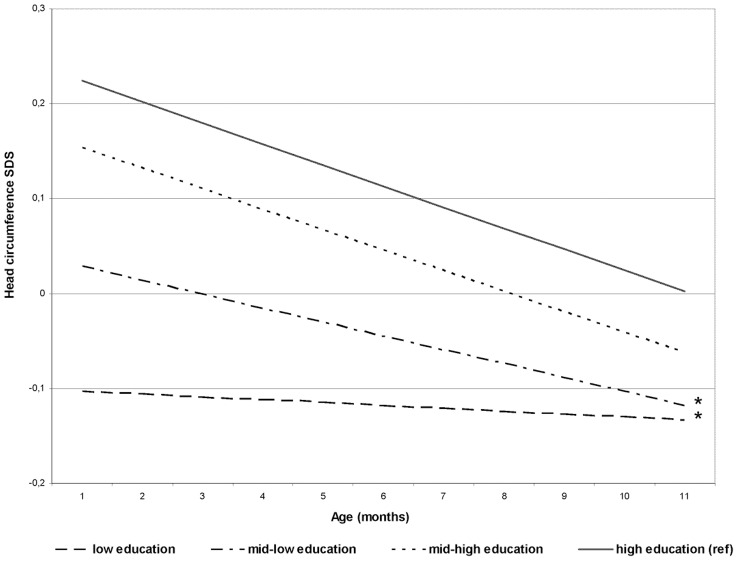
Association between maternal educational level and longitudinally measured head circumference growth^a^. ^ a^Results are based on linear mixed models and reflect the standard deviation scores of head circumference (based on 16958 measurements) growth in the first postnatal year in the offspring of mothers with low, mid-low, mid-high educational levels and high educational level. High education is reference group. *P for educational level *age ≤0.001.

Results from our linear mixed models showed that HC differences between the various educational subgroups became smaller with increasing age due to declining HC growth in the higher educational subgroups. Compared with children of mothers with high educational levels, those whose mothers had mid-low and low educational levels showed a relatively faster growth of head circumference (P for educational level * age ≤0.001) (Figure 2 and Table S3).

## Discussion

This study found children of mothers with a low and mid-low educational level to have significantly smaller HCs at the age of 1, 3 and 6 months compared to children of mothers with a high educational level. The factors which mainly explained the socioeconomic inequalities in HC were found to be birth weight, gestational age and the child’s length and weight.

The HC differences became smaller with increasing age. It has been suggested that infants whose intrauterine growth was constrained tend to grow faster postnatally to compensate [30], [31], [35]. Although we found HC differences to become smaller with age, this did not seem to be due to catch-up growth in the lower socioeconomic subgroup. We found that relative to the high socioeconomic subgroup, the low socioeconomic subgroup showed a continued high relative postnatal growth trajectory which reduced the difference between groups. One possible explanation is that children will grow to their genetic growth potential, which we assume is, on average, similar in all socioeconomic subgroups [36]. In the first months of life growth is largely a continuation of intrauterine growth, independent of genes, which appeared to be better in the higher socioeconomic subgroups [4], [31], [36]. Thus, one would expect HCs of children from higher subgroups to be larger compared to children from lower subgroups in the first months. With increasing age, when genetic factors may become more important, one would expect HC differences to decrease, because children grow to their genetic growth potential and the importance of genes are assumed to be equal for all socioeconomic subgroups [36]. Some studies found adults within lower socioeconomic subgroups to have smaller head sizes [37]. So, while our study showed marked inequalities in HC after birth, which declined during the first year, it is possible that inequalities in HC arise again later in life. For example, Gale et al. showed a relative increase in HC growth in the higher socioeconomic subgroup after the age of one [14].

In our study, the HC differences could mainly be explained by shorter gestational age and lower birth weight in the lower educational subgroups. At 1 and 3 months of age, the child’s weight and length only partially contributed to the HC differences. This suggests that socioeconomic inequalities in HC arise prenatally. It underlines the importance of preventing inequalities of birth outcomes arising in pregnancy, as they influence not only fetal [4], but also postnatal HC.

The potential mediators included in this study partially explained the educational differences in HC at 1 and 3 months. The remaining effect may be due to other factors, such as environmental and genetic factors [31], [36]. One such factor might be size and shape of the mother’s bony pelvis, since it has been reported that women with flat pelvises tend to have smaller babies with a smaller HC [38]. Flat pelvis is more common in women who have short stature and poor general physique which is more likely to appear among women with low SEP [32], [38]. Parental HC has been found to be a predictor of neonatal HC [8]. However, we did not have data on HC of the parents. This merits further investigation.

Parental height also contributed to HC differences between the different socioeconomic subgroups. The effect of parental height was strongest at 6 months of age. This is in agreement with the study of Smit et al., who found that heritability of head size is very low or absent in infants younger than 3 months and heritability estimates were 90% at 4 to 5 months [36]. Maternal height seemed to be of more importance on growth of HC than paternal height, which is in line with other studies [31].

Smoking during pregnancy contributed to larger HC differences at 6 months. This is in line with other studies which found impaired postnatal growth of infants prenatally exposed to cigarette smoking [31]. However, it is unclear why we only found an effect of smoking at the age of 6 months. Our findings confirm that reducing smoking rates among pregnant women is very important, since smoking not only impairs fetal HC growth [4], but also postnatal HC growth. Creating awareness among pregnant women, e.g. through midwifes, that smoking might be associated with smaller brain volume of their children in infancy, might increase their motivation to stop smoking.

Our study also showed that lower educated subgroups have factors that have suppressive effects on a small HC. For instance, high maternal BMI was positively associated with HC. This finding was also observed for neonatal HC and maternal BMI [39]. However, high BMI represents risks to a pregnant woman and her unborn child, and is therefore not recommended [40], [41].

The differences in HC between the various maternal educational levels could not be explained by psychosocial factors, drinking alcohol during pregnancy, smoking during pregnancy in the first 3 months, or having gestational diabetes. Since these factors are found to be negatively associated with birth weight and gestational age, they might act on HC via indirect pathways [42], [43].

The study of Silva et al. showed that a lower maternal educational level was associated with slower fetal growth and this effect appeared to be strongest for fetal brain, although this was not significant [4]. Therefore, we additionally explored whether HC was proportional to length per educational subgroup at 1, 3, 6 and 11 months. Regression analysis adjusting for maternal age and parity was used to calculate mean HC SDS minus mean length SDS. At 11 months, we found that HC was relatively smaller in relation to length in the low education subgroup than in the high education subgroup (p<0.05) (Figure S1). However, conclusions must be drawn carefully, since this could also indicate ease of moving weight and long bones rather than brain and skull. Further research is needed.

### Methodological Considerations

The main strength of this study lies in its population-based prospective design, in which a large number of women were enrolled early in pregnancy, and in the fact that information on relevant potential confounders and mediators was available. Limitation of this type of design is the sensitivity to selection bias, information bias and residual confounding due to unmeasured covariates. The choice whether to consider a factor a confounder or a mediator was based on pre-existing knowledge about social and biological determinants of growth. It is not always a straightforward one, though, and is sometimes arbitrary. Another source of discussion when defining a factor as a mediator, is the causal relationship that is inferred between SEP and that factor. Because actual establishment of causality is only possible with experimental data, one cannot exclude the possibility that the association between SEP and the mediator is not causal.

Although there are other measures of SEP, we used maternal educational level as indicator of SEP. Education is an important determinant of employment and economic circumstances, and thus reflects material resources. It also reflects non-economic social characteristics, such as general and health-related knowledge, which influences health behavior, literacy, problem-solving skills and prestige [15], [44]. Level of education has also been linked to a greater differentiation in health outcomes than other socioeconomic indicators [45]. Although educational level is a useful indicator of SEP, it may not entirely capture the material and financial aspects of SEP. Therefore, we repeated the analyses using household income level as determinant, and we found comparable results. There was one exception: income-related differences in HC were statistically significant at 11 months of age after adjustment for confounders. These HC differences could be explained with the same mediators as found at 6 months of age (data not shown).

To various extents, our results may have been influenced by the following limitations. Information on pre-pregnancy BMI, smoking and alcohol consumption during pregnancy and psychosocial determinants was derived from questionnaires. This may have induced some misclassification. Misclassification of potential mediating risk factors may have contributed to the lack of explanation of the observed association between maternal education and HC. Fetal growth restriction can lead to an underestimation of the effect of SEP on HC, because the gestation may be less certain for disadvantaged women if they have a higher proportion of unplanned pregnancies. Ultrasound correction of gestational age may then induce a bias if there is fetal growth restriction. In our study, however, there were no differences in the prevalence of children born with fetal growth restriction by educational subgroups (data not shown, available upon request). The response rate among Dutch pregnant women in The Generation R Study was relatively high (68%), but there was some selection towards a relatively highly educated and somewhat healthier study population, which may have led to some underestimation of the estimated effect of low maternal education [20]. Another possible limitation is that we excluded 770 children for several reasons. This could have led to selection bias. Finally, the relative impact of pre- and postnatal factors will depend on environmental conditions which may differ per country. The generalizability of our findings to other populations may therefore be limited.

### Conclusion

This study adds to the small body of literature concerning socioeconomic inequalities of HC in infancy.

Our results add to the evidence of the negative impact of a low SEP on different aspects of growth in childhood. These findings warrant a public health strategy aimed at tackling these inequalities, a strategy that should already start during the preconception period and should include the prevention of a low birth weight and short gestational age [46]. Furthermore, midwives, obstetricians and pediatricians should be aware of the impact of socioeconomic disadvantage on a child’s growth. Finally, our findings and the long-term consequences need to be confirmed in other studies.

## Supporting Information

Figure S1 **Mean head circumference SDS minus mean length SDS, stratified by maternal educational level.** SDS  =  standard-deviation score *Mean head circumference SDS - mean length SDS is significant different in the low educational subgroup from that in the subgroup with high education at level p≤0.05. All values are adjusted for maternal age and parity.
(TIF)

Table S1 **General characteristics of the excluded population (n = 770)^a^.** BMI = body mass index, SDS = standard deviation scores, HC = head circumference.^ a^Values are percentages or means (SD) for the total excluded population. ^b^P-values are calculated with the Chi-square test for categorical variables and ANOVA for continuous variables compared to the variables of the total included population. Values are calculated with non-imputed data.
(DOC)

Table S2 **Change in head circumference differences (in SDS) for maternal educational level after adjustment for potential mediators.** B = effect estimate, CI = confidence interval, BMI = body mass index.^ a^ Change a, b and c represent the changes in effect estimates for mid-high, mid-low and low education relative to model 1 (includes confounders) after individual adjustment for potential mediators (100×(B _model 1_– B _model 1 with mediator_)/(B_ model 1_ )). The percentages in bold attenuate with ≥10%.
(DOC)

Table S3 **Longitudinal associations between maternal educational level and child’s head circumference^a^.** ^a^Values are based on linear mixed models (based on 16958 measurements) and reflect the difference in growth in standard deviation score (SDS) of head circumference per educational subgroup compared to the high subgroup, which is the reference group. ^b^P-value reflects the significance level of the estimate.
(DOC)

## References

[1] Tanner JM (1992). Growth as a measure of the nutritional and hygienic status of a population.. Horm Res.

[2] Bobak M, Kriz B, Leon DA, Danova J, Marmot M (1994). Socioeconomic factors and height of preschool children in the Czech Republic.. Am J Public Health.

[3] du Prel X, Kramer U, Behrendt H, Ring J, Oppermann H (2006). Preschool children's health and its association with parental education and individual living conditions in East and West Germany.. BMC Public Health.

[4] Silva LM, Jansen PW, Steegers EA, Jaddoe VW, Arends LR (2010). Mother's educational level and fetal growth: the genesis of health inequalities.. Int J Epidemiol.

[5] Cecil JE, Watt P, Murrie IS, Wrieden W, Wallis DJ (2005). Childhood obesity and socioeconomic status: a novel role for height growth limitation.. Int J Obes (Lond).

[6] Rona RJ, Chinn S, Florey CD (1985). Exposure to cigarette smoking and children's growth.. Int J Epidemiol.

[7] Bartholomeusz HH, Courchesne E, Karns CM (2002). Relationship between head circumference and brain volume in healthy normal toddlers, children, and adults.. Neuropediatrics.

[8] Ivanovic DM, Leiva BP, Perez HT, Olivares MG, Diaz NS (2004). Head size and intelligence, learning, nutritional status and brain development. Head, IQ, learning, nutrition and brain.. Neuropsychologia.

[9] Larroque B, Bertrais S, Czernichow P, Leger J (2001). School difficulties in 20-year-olds who were born small for gestational age at term in a regional cohort study.. Pediatrics.

[10] Jokela M, Batty GD, Deary IJ, Gale CR, Kivimaki M (2009). Low childhood IQ and early adult mortality: the role of explanatory factors in the 1958 British Birth Cohort.. Pediatrics.

[11] Ivanovic DM, Leiva BP, Perez HT, Almagia AF, Toro TD (2002). Nutritional status, brain development and scholastic achievement of Chilean high-school graduates from high and low intellectual quotient and socio-economic status.. Br J Nutr.

[12] Malina RM, Habicht JP, Martorell R, Lechtig A, Yarbrough C (1975). Head and chest circumferences in rural Guatemalan Ladino children, birth to seven years of age.. Am J Clin Nutr.

[13] Nagra SA, Gilani AH (1984). Longitudinal study on head circumference of Pakistani infants in different socioeconomic groups.. Arch Latinoam Nutr.

[14] Gale CR, O'Callaghan FJ, Godfrey KM, Law CM, Martyn CN (2004). Critical periods of brain growth and cognitive function in children.. Brain.

[15] Galobardes B, Shaw M, Lawlor DA, Lynch JW, Davey Smith G (2006). Indicators of socioeconomic position (part 1).. J Epidemiol Community Health.

[16] Berkman LF, Kawachi I Lynch J. Kaplan GA. Socioeconomic position.. Oxford: Oxford University Press.

[17] (1994). Van de Mheen H, Stronks K, Van den Bos J, Mackenbach JP. De relatie tussen sociaal-economische status en verschillende indicatoren voor gezondheid [in Dutch].. Rijswijk: Ministerie van WVC.

[18] Parker JD, Schoendorf KC, Kiely JL (1994). Associations between measures of socioeconomic status and low birth weight, small for gestational age, and premature delivery in the United States.. Ann Epidemiol.

[19] Jaddoe VW, van Duijn CM, van der Heijden AJ, Mackenbach JP, Moll HA (2010). The Generation R Study: design and cohort update 2010.. Eur J Epidemiol.

[20] Jaddoe VW, Mackenbach JP, Moll HA, Steegers EA, Tiemeier H (2006). The Generation R Study: Design and cohort profile.. Eur J Epidemiol.

[21] (2004). Statistics Netherlands. Allochtonen in Nederlands 2004.. Voorburg/Heerlen.

[22] Fredriks AM, van Buuren S, Jeurissen SE, Dekker FW, Verloove-Vanhorick SP (2003). Height, weight, body mass index and pubertal development reference values for children of Turkish origin in the Netherlands.. Eur J Pediatr.

[23] Fredriks AM, van Buuren S, Jeurissen SE, Dekker FW, Verloove-Vanhorick SP (2004). Height, weight, body mass index and pubertal development references for children of Moroccan origin in The Netherlands.. Acta Paediatr.

[24] Fredriks AM, van Buuren S, Burgmeijer RJ, Meulmeester JF, Beuker RJ (2000). Continuing positive secular growth change in The Netherlands 1955–1997.. Pediatr Res.

[25] (2007). Growth Analyzer 3.0, Dutch Growth Research Foundation, Rotterdam, the Netherlands.

[26] McNamee R (2003). Confounding and confounders.. Occup Environ Med 60: 227–234; quiz 164, 234.

[27] Bauman AE, Sallis JF, Dzewaltowski DA, Owen N (2002). Toward a better understanding of the influences on physical activity: the role of determinants, correlates, causal variables, mediators, moderators, and confounders.. Am J Prev Med.

[28] Geraedts EJ, van Dommelen P, Caliebe J, Visser R, Ranke MB (2011). Association between head circumference and body size.. Horm Res Paediatr.

[29] Kramer MS, Guo T, Platt RW, Vanilovich I, Sevkovskaya Z (2004). Feeding effects on growth during infancy.. J Pediatr.

[30] Ong KK, Preece MA, Emmett PM, Ahmed ML, Dunger DB (2002). Size at birth and early childhood growth in relation to maternal smoking, parity and infant breast-feeding: longitudinal birth cohort study and analysis.. Pediatr Res.

[31] Hindmarsh PC, Geary MP, Rodeck CH, Kingdom JC, Cole TJ (2008). Factors predicting ante- and postnatal growth.. Pediatr Res.

[32] Galobardes B, McCormack VA, McCarron P, Howe LD, Lynch J (2012). Social inequalities in height: persisting differences today depend upon height of the parents.. PLoS One.

[33] MacKinnon DP, Krull JL, Lockwood CM (2000). Equivalence of the mediation, confounding and suppression effect.. Prev Sci.

[34] Greenland S, Finkle WD (1995). A critical look at methods for handling missing covariates in epidemiologic regression analyses.. Am J Epidemiol.

[35] Hokken-Koelega AC, De Ridder MA, Lemmen RJ, Den Hartog H, De Muinck Keizer-Schrama SM (1995). Children born small for gestational age: do they catch up?. Pediatr Res.

[36] Smit DJ, Luciano M, Bartels M, van Beijsterveldt CE, Wright MJ (2010). Heritability of head size in Dutch and Australian twin families at ages 0–50 years.. Twin Res Hum Genet.

[37] Tisserand DJ, Bosma H, Van Boxtel MP, Jolles J (2001). Head size and cognitive ability in nondemented older adults are related.. Neurology.

[38] Martyn CN, Barker DJ, Osmond C (1996). Mothers' pelvic size, fetal growth, and death from stroke and coronary heart disease in men in the UK.. Lancet.

[39] Voigt M, Rochow N, Jahrig K, Straube S, Hufnagel S (2010). Dependence of neonatal small and large for gestational age rates on maternal height and weight–an analysis of the German Perinatal Survey.. J Perinat Med.

[40] Yu CK, Teoh TG, Robinson S (2006). Obesity in pregnancy.. BJOG.

[41] Basso O, Rasmussen S, Weinberg CR, Wilcox AJ, Irgens LM (2006). Trends in fetal and infant survival following preeclampsia.. JAMA.

[42] Jansen PW, Tiemeier H, Jaddoe VW, Hofman A, Steegers EA (2009). Explaining educational inequalities in preterm birth: the generation r study.. Arch Dis Child Fetal Neonatal Ed.

[43] Jansen PW, Tiemeier H, Looman CW, Jaddoe VW, Hofman A (2009). Explaining educational inequalities in birthweight: the Generation R Study.. Paediatr Perinat Epidemiol.

[44] Braveman PA, Cubbin C, Egerter S, Chideya S, Marchi KS (2005). Socioeconomic status in health research: one size does not fit all.. JAMA.

[45] Winkleby MA, Jatulis DE, Frank E, Fortmann SP (1992). Socioeconomic status and health: how education, income, and occupation contribute to risk factors for cardiovascular disease.. Am J Public Health.

[46] Atrash H, Jack BW, Johnson K (2008). Preconception care: a 2008 update.. Curr Opin Obstet Gynecol.

