# Supplementary material for: Head Circumference of Infants Born to Mothers with Different Educational Levels; The Generation R Study
Source: PLoS One. 2012 Jun 29;7(6):e39798. doi: 10.1371/journal.pone.0039798 (PMC3387269; doi:10.1371/journal.pone.0039798)
Supplement: Table S1 — General characteristics of the excluded population (n = 770)a. BMI = body mass index, SDS = standard deviation scores, HC = head circumference. aValues are percentages or means (SD) for the total excluded population. bP-values are calculated with the Chi-square test for categorical variables and ANOVA for continuous variables compared to the variables of the total included population. Values are calculated with non-imputed data. (DOC) [file pone.0039798.s002.doc]

**Table S1. General characteristics of the excluded population (n = 770)**a

|  | **Total n=770** | **P-value^b^** |
| --- | --- | --- |
| **Pregnancy and birth characteristics** |  |  |
| Maternal age (years) | 32.7 (4.0) | <0.001 |
| Parity (% nullipara) | 21.3% | <0.001 |
| Infant gender (% girls) | 48.3% | 0.456 |
| Gestational age at birth (weeks) | 38.8 (2.7) | <0.001 |
| Birth weight (grams) | 3196.5 (805.4) | <0.001 |
| Gestational diabetes (% yes) | 1.0 | 0.576 |
| Maternal smoking during pregnancy (%) |  |  |
| None | 82.0 | 0.007 |
| Maternal alcohol use during pregnancy (%) |  |  |
| None | 33.4 | 0.626 |
| **Parental anthropometrics** |  |  |
| Maternal height (cm) | 170.9 (6.8) | 0.081 |
| Pre-pregnancy BMI mother (kg/m^2^) | 23.2 (3.6) | 0.884 |
| Paternal height (cm) | 184.1 (7.4) | 0.930 |
| **Psychosocial and material factors** |  |  |
| Financial difficulties (% yes) | 14.0 | 0.352 |
| Pregnancy planned (% no) | 18.9 | 0.898 |
| **Child characteristics** |  |  |
| Length SDS at 1 month of age | -0.31 (1.3) | 0.006 |
| Length SDS at 3 months of age | -0.15 (1.1) | 0.002 |
| Length SDS at 6 months of age | -0.10 (1.0) | 0.058 |
| Length SDS at 11 months of age | -0.20 (1.0) | 0.189 |
| Weight SDS at 1 month of age | 0.03 (1.4) | 0.002 |
| Weight SDS at 3 months of age | 0.05 (1.2) | 0.002 |
| Weight SDS at 6 months of age | -0.07 (1.0) | 0.002 |
| Weight SDS at 11 months of age | -0.14 (1.0) | 0.011 |
| HC SDS at 1 month of age | 0.03 (1.1) | 0.002 |
| HC at 3 months of age | -0.10 (0.9) | 0.003 |
| HC at 6 months of age | -0.13 (0.9) | 0.180 |
| HC at 11 months of age | -0.09 (0.9) | 0.431 |
| Breastfeeding (yes %) | 88.7 | 0.430 |
