# Supplementary material for: Head Circumference of Infants Born to Mothers with Different Educational Levels; The Generation R Study
Source: PLoS One. 2012 Jun 29;7(6):e39798. doi: 10.1371/journal.pone.0039798 (PMC3387269; doi:10.1371/journal.pone.0039798)
Supplement: Table S2 — Change in head circumference differences (in SDS) for maternal educational level after adjustment for potential mediators. B = effect estimate, CI = confidence interval, BMI = body mass index. a Change a, b and c represent the changes in effect estimates for mid-high, mid-low and low education relative to model 1 (includes confounders) after individual adjustment for potential mediators (100×(B model 1– B model 1 with mediator)/(B model 1 )). The percentages in bold attenuate with ≥10%. (DOC) [file pone.0039798.s003.doc]

**Table S2.** **Change in head circumference differences (in SDS) for maternal educational level after adjustment for potential mediators**

| **Level of maternal education** | **High (n=1122)** | **Mid-high (n=871) B (95% CI)** | **Change a^a^** | **Mid-low (n=895) B (95% CI)** | **Change b^a^** | **Low (n=495) B (95% CI)** | | **Change c^a^** |
| --- | --- | --- | --- | --- | --- | --- | --- | --- |
| **1 month** |  |  |  |  | | | | |
| **Model 1** (includes maternal age and parity) | Reference | -0.07 (-0.17,0.02) |  | -0.17 (-0.27,-0.07) |  | -0.42 (-0.54,-0.30) | |  |
| **Pregnancy characteristics** |  |  |  |  |  |  | |  |
| Model 1 + birth weight SDS | Reference | -0.05 (-0.13,0.03) | **-31.1%** | -0.10 (-0.18,-0.01) | **-56.2%** | -0.27 (-0.38,-0.16) | | **-35.5%** |
| Model 1 + gestational age | Reference | -0.05 (-0.13,0.03) | **-27.0%** | -0.11 (-0.19, -0.03) | **-34.9%** | -0.30 (-0.40,-0.19) | | **-28.8%** |
| Model 1 + gestational diabetes | Reference | -0.07 (-0.17,0.02) | -1.4% | -0.17 (-0.27,-0.07) | 0% | -0.42 (-0.54,-0.30) | | 0% |
| Model 1+ smoking during pregnancy | Reference | -0.07 (-0.16,0.03) | -8.1% | -0.15 (-0.25,-0.06) | -9.5% | -0.38 (-0.50,-0.25) | | -9.8% |
| Model 1 + alcohol consumption during pregnancy | Reference | -0.08 (-0.17,0.02) | +1.4% | -0.17 (-0.27,-0.07) | +2.4% | -0.42 (-0.54,-0.30) | | +1.2% |
| **Parental anthropometrics** |  |  |  |  |  |  | |  |
| Model 1 + maternal height | Reference | -0.07 (-0.16,0.02) | -6.8% | -0.14 (-0.24,-0.04) | **-17.8%** | - 0.35 (-0.47,-0.23) | | **-16.1%** |
| Model 1 + paternal height | Reference | -0.06 (-0.15,0.04) | **-22.9%** | -0.15 (-0.24,-0.05) | **-13.0%** | -0.37 (-0.49,-0.23) | | **-10.8%** |
| Model 1 + pre-pregnancy BMI | Reference | -0.08 (-0.17,0.02) | +5.4% | -0.19 (-0.29,-0.09) | **+11.2%** | -0.44 (-0.57,-0.32) | | +6.5% |
| **Psychosocial and material factors** |  |  |  |  |  |  | |  |
| Model 1 + pregnancy planned | Reference | -0.07 (-0.17,0.02) | -2.7% | -0.17 (-0.26,-0.07) | -2.4% | -0.41 (-0.53,-0.29) | | -2.2% |
| Model 1 + financial difficulties | Reference | -0.07 (-0.16,0.02) | -4.1% | -0.16 (-0.26,-0.07) | -3.6% | -0.41 (-0.53,-0.28) | | -2.6% |
| **Child determinants** |  |  |  |  |  |  | |  |
| Model 1 + child’s weight SDS at 1 month | Reference | -0.06 (-0.13,0.01) | **-24.3%** | -0.07 (-0.15,-0.002) | **-56.2%** | -0.16 (-0.25,-0.07) | | **-62.8%** |
| Model 1 + child’s height SDS at 1 month | Reference | -0.05 (-0.13,0.03) | **-32.4%** | -0.10 (-0.19,-0.02) | **-39.6%** | -0.22 (-0.32,-0.11) | | **-47.5%** |
| Model 1 + breastfeeding (yes/no) | Reference | -0.07 (-0.16,0.03) | -9.5% | -0.15 (-0.25,-0.05) | **-10.7%** | -0.38 (-0.50,-0.26) | | -8.6% |
| **3 months** |  |  |  |  |  | |  |  |
| **Model 1** (includes maternal age and parity) | Reference | -0.10 (-0.19,-0.01) |  | -0.11 (-0.21,-0.02) |  | | -0.27 (-0.40,-0.15) |  |
| **Pregnancy characteristics** |  |  |  |  |  |  | |  |
| Model 1 + birth weight SDS | Reference | -0.10 (-0.18,-0.01) | -3.1% | -0.08 (-0.17,0.01) | **-26.5%** | -0.18 (-0.29,-0.07) | | **-34.2%** |
| Model 1 + gestational age | Reference | -0.08 (-0.17,0.002) | **-16.3%** | -0.08 (-0.17,0.01) | **-31.9%** | -0.20 (-0.31,-0.10) | | **-25.0%** |
| Model 1 + gestational diabetes | Reference | -0.10 (-0.19,-0.01) | -1.0% | -0.11 (-0.21,-0.02) | -0.9% | -0.27 (-0.39,-0.16) | | +0.7% |
| Model 1+ smoking during pregnancy | Reference | -0.10 (-0.19,-0.003) | -3.1% | -0.11 (-0.20,-0.01) | -5.3% | -0.26 (-0.38,-0.13) | | -5.9% |
| Model 1 + alcohol consumption during pregnancy | Reference | -0.10 (-0.19,-0.01) | +1.0% | -0.12 (-0.21,-0.02) | +3.5% | -0.28 (-0.40,-0.16) | | +1.8% |
| **Parental anthropometrics** |  |  |  |  |  |  | |  |
| Model 1 + maternal height | Reference | -0.09 (-0.18,-0.003) | -4.1% | -0.09 (-0.18,0.01) | **-21.2%** | -0.22 (-0.34,-0.10) | | **-19.1%** |
| Model 1 + paternal height | Reference | -0.09 (-0.18,0.004) | **-12.2%** | -0.10 (-0.20,-0.01) | **-10.6%** | -0.24 (-0.35,-0.12) | | **-13.2%** |
| Model 1 + pre-pregnancy BMI | Reference | -0.10 (-0.19,-0.01) | +1.0% | -0.13 (-0.22,-0.03) | **+11.5%** | -0.29 (-0.41,-0.17) | | +7.7% |
| **Psychosocial and material factors** |  |  |  |  |  |  | |  |
| Model 1 + pregnancy planned | Reference | -0.10 (-0.19,-0.01) | 0% | -0.12 (-0.21,-0.02) | +1.8% | -0.28 (-0.40,-0.16) | | +1.5% |
| Model 1 + financial difficulties | Reference | -0.10 (-0.19, -0.003) | -3.1% | -0.11 (-0.21,-0.01) | -3.5% | -0.26 (-0.38,-0.14) | | -3.3% |
| **Child determinants** |  |  |  |  |  |  | |  |
| Model 1 + child’s weight SDS at 3 months | Reference | -0.09 (-0.17,-0.02) | -6.1% | -0.10 (-0.17,-0.02) | **-14.2%** | -0.19 (-0.28,-0.09) | | **-31.3%** |
| Model 1 + child’s height SDS at 3 months | Reference | -0.06 (-0.14,0.02) | **-37.8%** | -0.08 (-0.16,0.01) | **-31.0%** | -0.19 (-0.30,-0.08) | | **-30.5%** |
| Model 1 + breastfeeding (yes/no) | Reference | -0.9 (-0.19,-0.003) | -4.1% | -0.10 (-0.20,-0.003) | **-12.4%** | -0.25 (-0.37,-0.13) | | 8.8% |
| **6 months** |  |  |  |  | | | | |
| **Model 1** (includes maternal age and parity) | Reference | -0.06 (-0.15,0.02) |  | -0.11 (-0.19,-0.02) |  | -0.13 (-0.24,-0.02) | |  |
| **Pregnancy characteristics** |  |  |  |  |  |  | |  |
| Model 1 + birth weight SDS | Reference | -0.05 (-0.13,0.03) | **-19.0%** | -0.07 (-0.15,0.02) | **-39.3%** | -0.02 (-0.13,0.08) | | **-81.4%** |
| Model 1 + gestational age | Reference | -0.05 (-0.13,0.03) | **-17.5%** | -0.07 (-0.15,0.01) | **-34.6%** | -0.07 (-0.18,0.03) | | **-42.6%** |
| Model 1 + gestational diabetes | Reference | -0.06 (-0.15,0.02) | 0% | -0.11 (-0.19,-0.02) | -0.9% | -0.13 (-0.24,-0.02) | | +1.6% |
| Model 1+ smoking during pregancy | Reference | -0.06 (-0.14,0.02) | -4.8% | -0.10 (-0.18,-0.01) | -7.5% | -0.11 (-0.22,0.002) | | **-16.2%** |
| Model 1 + alcohol consumption during pregnancy | Reference | -0.07 (-0.15,0.02) | +3.2% | -0.11 (-0.20,-0.02) | +1.9% | -0.13 (-0.24,-0.02) | | +2.3% |
| **Parental anthropometrics** |  |  |  |  |  |  | |  |
| Model 1 + maternal height | Reference | -0.06 (-0.17,0.04) | -6.3% | -0.08 (-0.17,0.002) | **-23.4%** | -0.06 (-0.17,0.04) | | **-50.4%** |
| Model 1 + paternal height | Reference | -0.05 (-0.13,0.03) | -19.0% | -0.09 (-0.17,-0.01) | **-15.9%** | -0.09 (-0.20,0.01) | | **-27.9%** |
| Model 1 + pre-pregnancy BMI | Reference | -0.07 (-0.15,0.02) | +3.2% | -0.12 (-0.21,-0.03) | **+11.2%** | -0.15 (-0.25,-0.04) | | **+13.2%** |
| **Psychosocial and material factors** |  |  |  |  |  |  | |  |
| Model 1 + pregnancy planned | Reference | -0.06 (-0.15,0.02) | 0% | -0.11 (-0.19,-0.02) | 0% | -0.13 (-0.24,-0.02) | | -0.8% |
| Model 1 + financial difficulties | Reference | -0.06 (-0.15,0.02) | 0% | -0.11 (-0.19,-0.02) | -0.9% | -0.13 (-0.24,-0.02) | | -1.6% |
| **Child determinants** |  |  |  |  |  |  | |  |
| Model 1 + child’s weight SDS at 6 months | Reference | -0.05 (-0.12,0.03) | **-28.6%** | -0.10 (-0.17,-0.03) | -8.4% | -0.12 (-0.21,-0.03) | | -8.5% |
| Model 1 + child’s height SDS at 6 months | Reference | -0.04 (-0.12,0.03) | **-30.2%** | -0.10 (-0.18,-0.02) | -6.5% | -0.12 (-0.22,-0.02) | | -7.8% |
| Model 1 + breastfeeding (yes/no) | Reference | -0.06 9-0.15,0.02) | -1.6% | -0.10 (-0.19,-0.02) | -4.7% | -0.12 (-0.23,-0.01) | | -6.2% |
