# Supplementary material for: Head Circumference of Infants Born to Mothers with Different Educational Levels; The Generation R Study
Source: PLoS One. 2012 Jun 29;7(6):e39798. doi: 10.1371/journal.pone.0039798 (PMC3387269; doi:10.1371/journal.pone.0039798)
Supplement: Table S3 — Longitudinal associations between maternal educational level and child’s head circumferencea. aValues are based on linear mixed models (based on 16958 measurements) and reflect the difference in growth in standard deviation score (SDS) of head circumference per educational subgroup compared to the high subgroup, which is the reference group. bP-value reflects the significance level of the estimate. (DOC) [file pone.0039798.s004.doc]

**Table S3. Longitudinal associations between maternal educational level and child’s head circumference**a

|  | **Difference in growth rate of head circumference** | | | |
| --- | --- | --- | --- | --- |
| **Maternal educational level** | **Intercept** | **P-value^b^** | **Slope (SDS (95% CI))** | **P-value^b^** |
| High | 0.2467 | <0.001 | Reference |  |
| Mid-high | 0.1756 | 0.069 | 0.001 (-0.003,0.005) | 0.767 |
| Mid-low | 0.0433 | <0.001 | 0.008(0.003,0.012) | <0.001 |
| Low | -0.1000 | <0.001 | 0.019(0.014,0.024) | <0.001 |
